# Prenatal Diagnosis of Cantrell's Pentalogy Associated with Agenesis of Left Limb in a Twin Pregnancy

**DOI:** 10.1155/2014/314284

**Published:** 2014-03-23

**Authors:** Yigit Cakiroglu, Emek Doger, Sule Yildirim Kopuk, Kadir Babaoglu, Eray Caliskan, Gulseren Yucesoy

**Affiliations:** Department of Obstetrics and Gynecology, Kocaeli University School of Medicine, Umuttepe Kampusu, Kocaeli, Turkey

## Abstract

Pentalogy of Cantrell is a rare malformation described in the literature. We report a case of pentalogy of Cantrell associated with left limb deficiency in a twin pregnancy. The fetus with multiple anomalies revealed kyphosis, ectopia cordis, and a large defect with protruding liver and bowel loops at 12 weeks and 3 days of gestational age on ultrasound scan. The other fetus was ultrasonographically normal. We diagnosed a case of pentalogy of Cantrell in a twin pregnancy after exclusion of limb body wall complex, body stalk anomaly, and amniotic band syndrome and after delivery of the fetuses. Macroscopic examinations were ectopia cordis, extrusion of the abdominal organs without membranes surrounding, and agenesis of the left limb.

## 1. Introduction

Many cases of pentalogy of Cantrell (PC) have been reported since the first case which was defined by Cantrell and colleagues in 1958 [1]. The initial syndrome was diagnosed as a pentad including midline supraumbilical abdominal wall defects, deficiency of the anterior diaphragm, defects of the lower sternum, defects of the diaphragmatic pericardium, and congenital intracardiac abnormalities. Variable cardiac anomalies have been reported and among the anomalies, ventricular septal defects, atrial septal defects, or other abnormalities are the most common reported.

We have reviewed the published literature on PC and reported a twin case with the diagnosis of PC and a normal fetus.

## 2. Case Report

A 20-year-old gravida 1, para 0, abortus 0 pregnant woman was admitted to our perinatology unit with the suspicion of ectopia cordis in a twin pregnancy at the 12th week of gestation.

**Figure 1:**
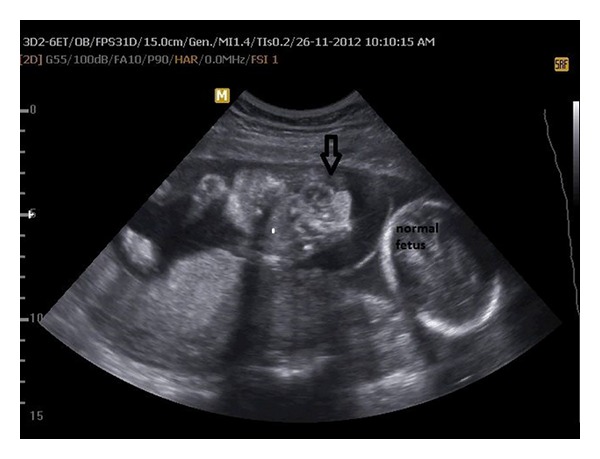
Ultrasonographic view of twins at 23 weeks of gestational age; arrow indicated heart protruding out of the sternal defect.

**Figure 2:**
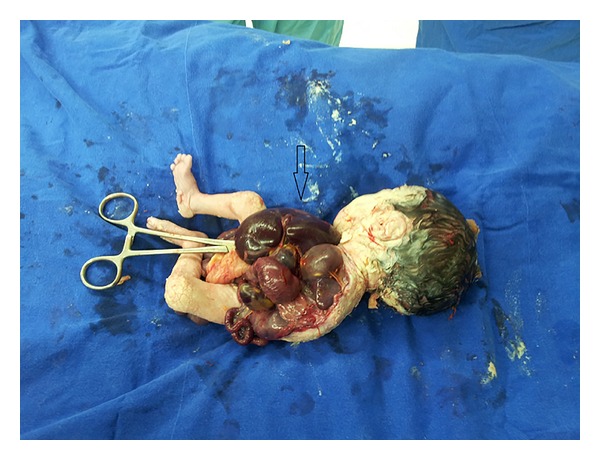
Postmortem photo of case showed large omphalocele with evisceration of all the gastrointestinal organs with absent sternum and rib cage and left upper limb are not shown.

She was performed transvaginal ultrasound scan and the scan revealed a dichorionic-diamniotic twin pregnancy. Crown-rump lengths were 48 mm and 46 mm corresponding to 11 + 5 weeks and nuchal translucencies were 1.2 and 1.6 mm, respectively. Ultrasound scan of the first twin revealed ectopia cordis and a large defect with protruding liver and bowel loops (Figure 1). The second twin was ultrasonographically normal. The diagnosis of PC was suspected after exclusion of limb body wall complex (LBWC), amniotic band syndrome, and body stalk anomaly. The parents were informed about the syndrome and the prognosis and were offered selective feticide procedure. They chose to continue the pregnancy without any intervention. A 1–3 apgared 1000 gr female fetus and a 7-8 apgared 2500 gr male fetus were born by a cesarean section at 37 weeks of gestation. The macroscopic observations were ectopia cordis, extrusion of the abdominal organs without membranes surrounding, hypoplastic ribs, sternum, and agenesis of the left limb (Figure 2). The second twin was macroscopically normal without any malformations. The family refused an autopsy procedure.

## 3. Discussion

Pentalogy of Cantrell is a rare syndrome with an estimated incidence of 5.5 per 1 million live births [2]. The etiology has not been fully determined but the most accepted hypothesis is developmental failure of a segment of the lateral mesoderm at around 14–18 days of embryonic life [1]. The full spectrum consists of midline supraumbilical abdominal wall defects, deficiency of the anterior diaphragm, defects of the lower sternum, defects of the diaphragmatic pericardium, and congenital intracardiac anomalies.

Various intracardiac anomalies have been described in the PC including ventricular septal defects (VSD) (100%), atrial septal defects (ASD) (53%), tetralogy of Fallot (TOF) (20%), pulmonary stenosis (33%), and left ventricular diverticulum (20%), whereas Vazquez-Jimenez et al. have reported that VSD is not consistent in every case (72%) [1, 3]. Likewise, VSD was not diagnosed in our case. Also, craniofacial and central nervous system anomalies, limb defects such as clubfoot, tibia, and radius agenesis, hypodactyly, and hypoplasia are the other associated anomalies [4, 5].

PC is a syndrome characterized by a distinct phenotypic variability. Toyama suggested three classes of PC: class 1: complete form, all five defects are present; Class 2: probable diagnosis that four defects are present, including intracardiac and ventral wall abnormalities; and class 3: incomplete expression, always including sternal abnormality with various combinations of defects [6]. Class 1-complete form of PC- has the worst prognosis and intracardiac defects which do not influence prognosis [7]. However, Grethel et al. have reported a case report with severe intracardiac malformations that has poor prognosis [8].

Abdominal wall defects that are seen in PC are omphalocele, diastasis recti, epigastric hernia, umblical hernia, and combined defects. Sternal malformations that can be seen in PC include bifid sternum, absent xiphoid process, short sternum, and defective formation of lower third [9]. Differential diagnosis of midline defects includes PC, LBWC, body stalk anomaly, and amniotic band syndrome.

We have reviewed the published literature on PC and reported a twin case with the diagnosis of PC and a normal fetus. Uygur et al. and Chen et al. have reported two cases with pentalogy of Cantrell and limb defects [4, 10]. Chen et al. reported a twin case with one of the fetuses having concomitant anencephaly and left upper limb hypoplasia [11].

PC is a syndrome with high mortality rate and the survival rate is lower than 40% [12]. The optimal management strategies and prognostic indices for neonates remain to be established. Generally, most of the fetuses associated with ectopia cordis indicate poor prognosis, whereas ectopia cordis in partial association with incomplete presentation of PC is likely more favorable [13].

In conclusion, early counseling should be made to make differential diagnosis. When the PC is diagnosed, a multidisciplinary team including an obstetrician, a neonatologist, a pediatric cardiologist, a pediatric surgeon, and a genetics specialist should agree on the best approach to PC.
